# Transcriptome and proteome analysis of the antitumor activity of maslinic acid against pancreatic cancer cells

**DOI:** 10.18632/aging.203623

**Published:** 2021-10-12

**Authors:** Hewei Zhang, Lijun Kong, Yan Zhang, Cheng Wang, Linxiao Sun

**Affiliations:** 1 Department of Surgery, Key Laboratory of Diagnosis and Treatment of Severe Hepato-Pancreatic Diseases of Zhejiang Province, Zhejiang Provincial Top Key Discipline in Surgery, The First Affiliated Hospital of Wenzhou Medical University, Wenzhou, Zhejiang, P.R. China

**Keywords:** maslinic acid, pancreatic cancer, malignant behavior, transcriptomics, proteomics

## Abstract

Maslinic acid (MA) is a triterpenoid compound of natural abundance in olive plants possessing numerous biological activities. The effect and molecular mechanism of MA on pancreatic cancer cells remain elusive. Here, we explored the anti-tumor activity of MA on human pancreatic cancer cells and the potential underlying molecular mechanism. The anti-cancer effects of MA on whole-cell processes, including proliferation, migration, and invasion in pancreatic cancer cells, were systematically assessed by colony formation, transwell, and migration assays. The search for potential therapeutic targets was achieved via transcriptomics and proteomics analyses. MA was demonstrated to inhibit the proliferation, migration, and invasion of PANC-1 and Patu-8988 cells, but induced apoptosis of these cells. Several key candidate genes and proteins of functional relevance for the anti-tumor activity of MA were identified through the association analysis of transcriptomics and proteomics. To our knowledge, this is the first transcription and proteomics-based comprehensive analysis of the mechanism of MA against pancreatic cancer. The findings demonstrate that MA holds promise as a therapeutic drug for managing pancreatic cancer.

## INTRODUCTION

Pancreatic cancer is a highly lethal malignancy with a poor prognosis due to its high propensity for local invasion and early metastasis [1]. While there are viable attempts to improve the detection and treatment of pancreatic cancer, the overall five-year survival rate is low, approximated at 6% [2]. Gemcitabine and 5-fluorouracil, first-line chemotherapeutic agents, have been widely adopted to manage pancreatic cancer; however, drug resistance of cancer cells greatly limits their efficacy [3]. Therefore, new anti-cancer drugs are needed urgently to improve the therapeutic effects and survival of pancreatic cancer patients.

Triterpenoids are a class of compounds, including oleanolic acid (OA) extracted from garlic and apple, ursolic acid (UA) extracted from blueberry and cranberry, succinic acid, and betulinic acid extracted from lavender, mistletoe, characterized by different structures [4]. Maslinic acid (MA) is also one of the triterpenoids, widely present in olives, mustard, basil, and hawthorn [5]. Previous evidence indicates that MA exerts anti-inflammatory, antibacterial, and antioxidant effects [6–8]. In recent years, emerging evidence has confirmed the association of MA with several types of cancer [9–11]. Studies have shown that MA can inhibit the migration, invasion, and adhesion of prostate cancer cells by suppressing the expression of hypoxia-inducible factor 1α (HIF-1α) [12]. MA also has potent significant inhibitory effects on the expression of B cell lymphoma 2 (Bcl-2) and elevate the expression of Bcl-2-associated X protein (Bax) to activate mitochondrial apoptosis pathway in colon cancer cells [13]. In this view, MA is hypothesized to have potential inhibitory effect on pancreatic cancer cells.

Here, the effects of MA on the proliferation, migration, invasion, and apoptosis of pancreatic cancer cells (PCCs) are comprehensively evaluated by real-time cell analysis, clonal formation, cell migration, and flow cytometry analyses. Several key genes and proteins associated with the anti-tumor activity of MA have been identified through transcriptomics and proteomics. The findings provide strong evidence that MA suppresses the growth, invasion, and migration of human pancreatic cancer and induces apoptosis by regulating some key genes. This work can provide basic data for further research on MA.

## RESULTS

### MA inhibits proliferation and induces apoptosis of PCCs

**Figure 1:**
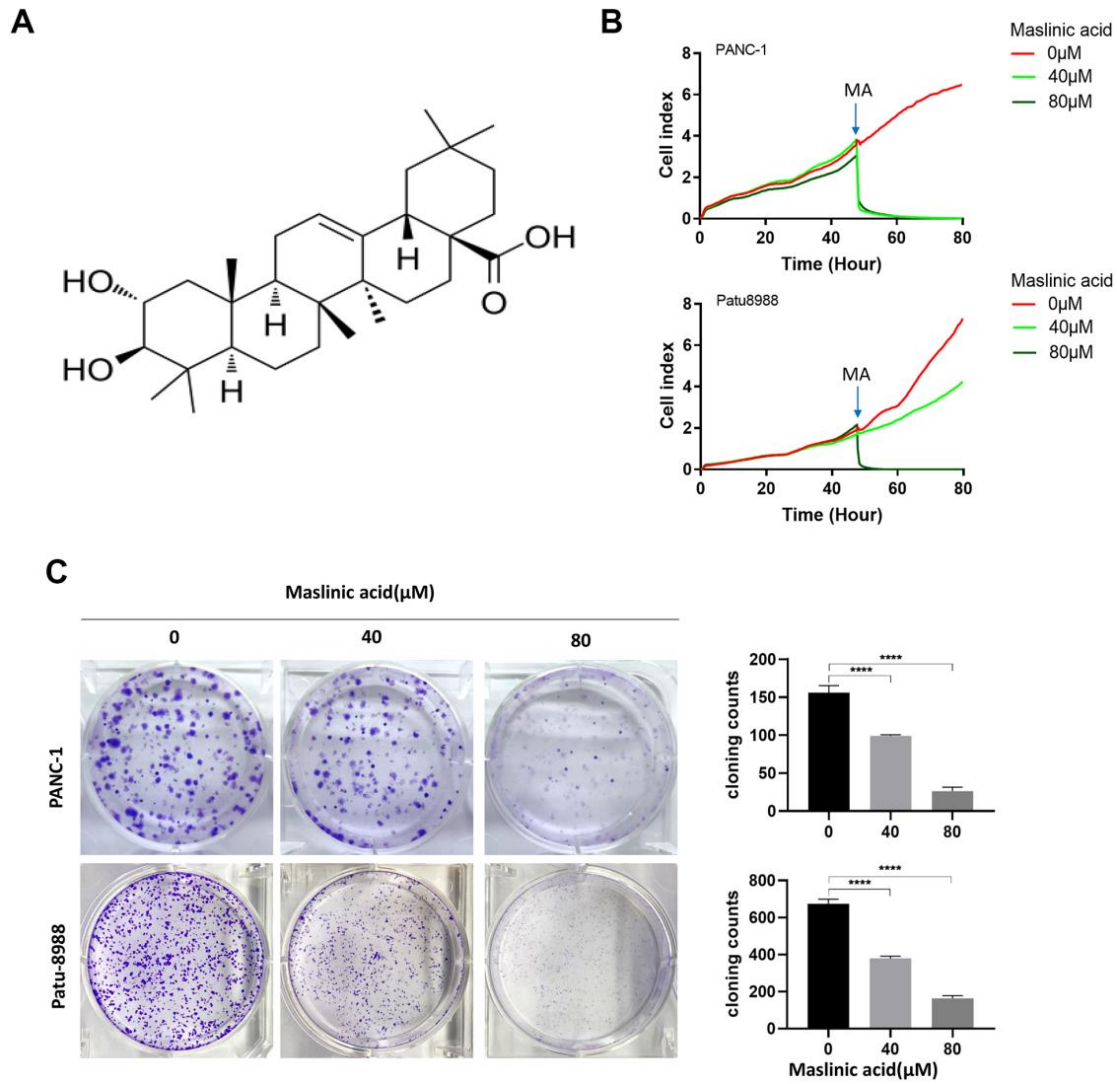
**Maslinic acid (MA)-induced inhibition of the proliferation of PANC-1 and Patu-8988 cells.** (**A**) The chemical structure of MA. (**B**) Label-free Real-time Cellular Analysis (RTCA) of PANC-1 and Patu-8988 cells incubated with MA (0, 40, 80 μM). (**C**) The effects of MA on the colony formation of PANC-1 and Patu-8988 cells. The cells were exposed to MA (0, 40, 80 μM) for 24 h. After 14 days cells were stained with crystal violet and colony counted. Data are representative of three independent experiments, expressed as mean ± SD. *p <0.05, **p < 0.01, ***p < 0.001, ****p < 0.0001.

**Figure 2:**
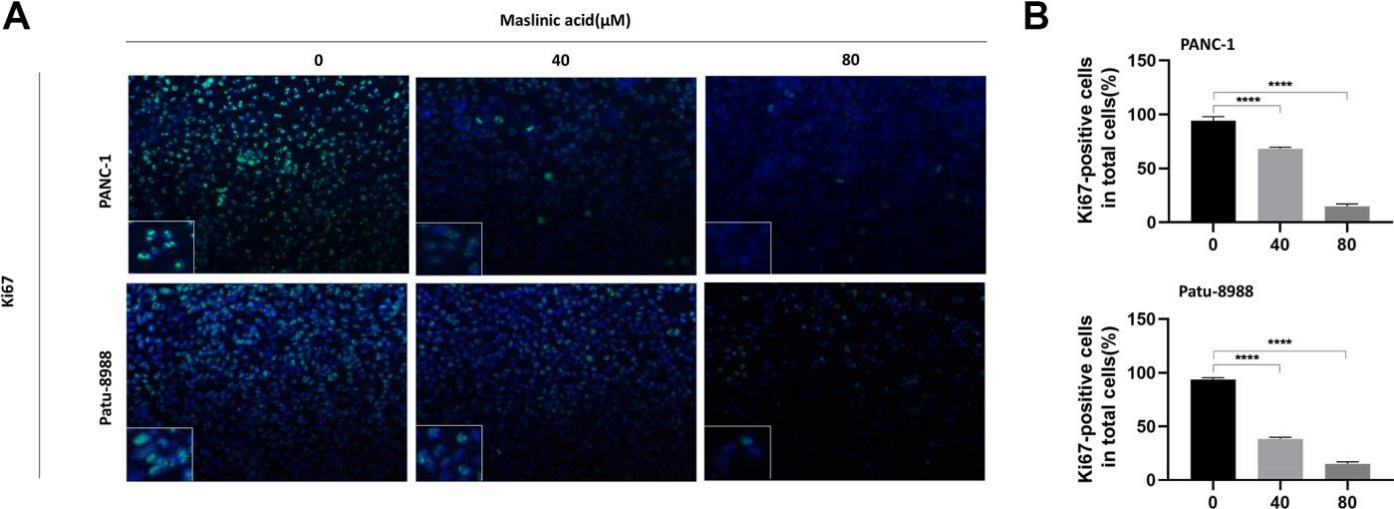
**MA-induced inhibition of the expression of ki67 protein of pancreatic cancer cells.** (**A**) Ki67 Immunofluorescence following PANC-1 and Patu-8988 cells incubation with MA (0, 40, 80 μM) for 24 h. Scale bar =50μm. (**B**) Quantitative analysis of Ki67 positive cells in different groups. Data are representative of three independents experiments and expressed as mean ± SD. ****p < 0.0001.

The effects of MA on the proliferation of PANC-1 and Patu-8988 cells were evaluated through unlabeled Real-Time Cell Analysis (RTCA), Ki67 immunofluorescence, and colony formation assay. RTCA showed that MA treatment (40 and 80 μM) significantly inhibited the proliferation of PANC-1 and Patu-8988 (Figure 1B) cells. Similar results as those of RTCA were demonstrated by colony formation assay (Figure 1C). Ki67 Immunofluorescence (Figure 2A) showed that the Ki67 green fluorescence in the MA-treated groups was reduced significantly compared to the control group (Figure 2B). These findings strongly demonstrate that MA treatment suppresses the proliferation of PANC-1 and Patu-8988 cells in a dose-dependent manner.

**Figure 3:**
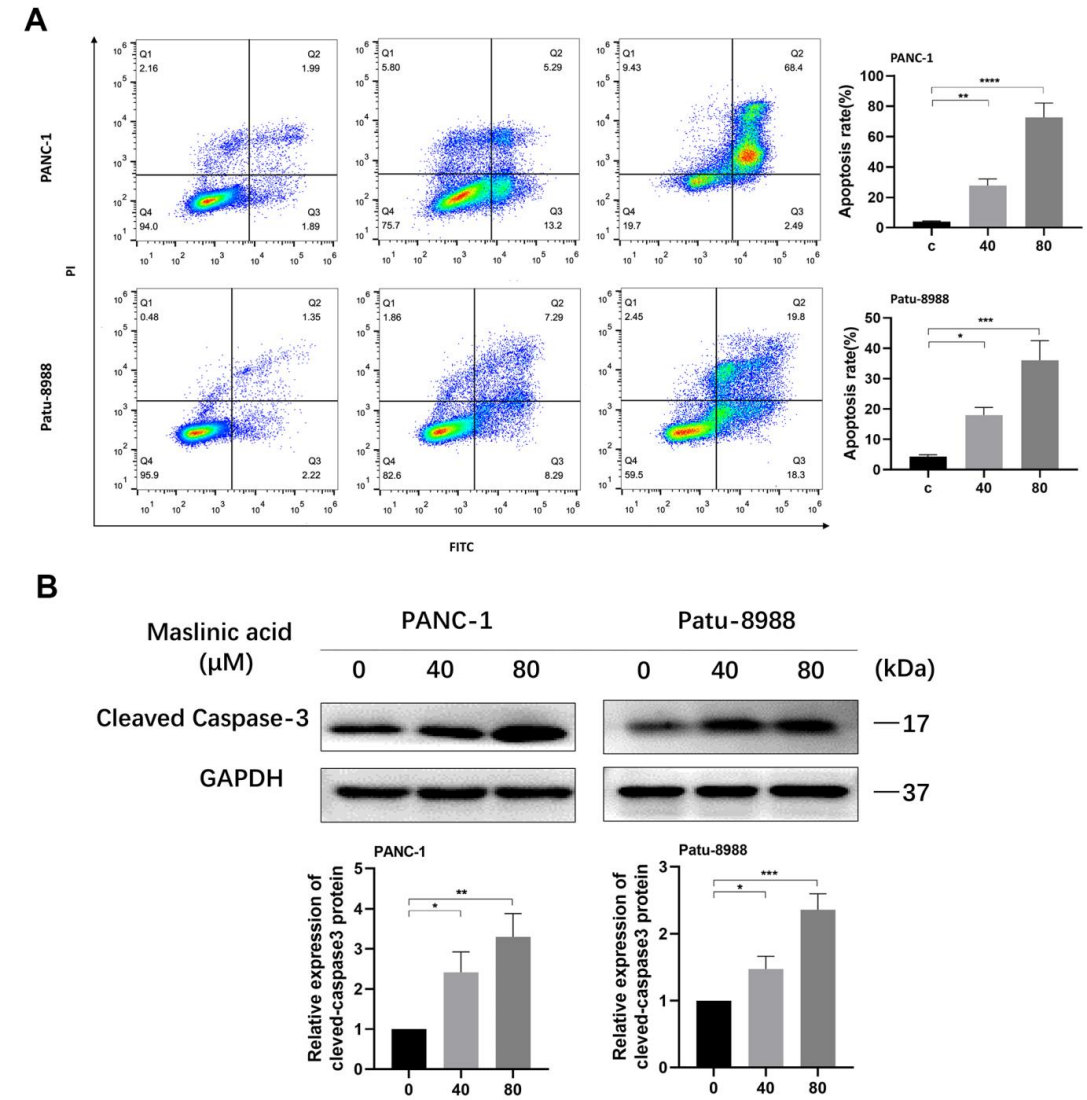
**MA-induced apoptosis of pancreatic cancer cells.** (**A**) Flow cytometry detection of the apoptosis of PANC-1 and Patu-8988 cells exposed to MA for 24 h, analyzed by Annexin V/PI staining. (**B**) Western blot analysis showing the expression of cleaved-caspase3 in MA-treated PANC-1 and Patu-8988 cells. Data are representative of three independents experiments and expressed as mean ± SD. *p <0.05, **p < 0.01, ***p < 0.001, ****p < 0.0001.

To investigate whether MA induces apoptosis of pancreatic cancer cells, PANC-1, and Patu-8988 cells were treated with MA at a concentration of 0, 40, and 80 μM for 24 h. The Annexin V-FITC/PI assay was used to assess apoptosis. Results demonstrated a considerable increase in the percentage of apoptotic cells compared with that of untreated cells, following MA treatment. The induction of apoptosis was increased from 3.88% to 18.49% in PANC-1 cells and from 3.57% to 16.58% in Patu-8988 cells (Figure 3A). Western blot analyses further confirmed the ability of MA in increasing cleaved caspase-3 expression levels in both PANC-1 and Patu-8988 cells (Figure 3B), supporting the view that MA treatment promotes apoptosis of PANC-1 and Patu-8988 cells in a dose-dependent manner.

### MA inhibits migration and invasion of PCCs

**Figure 4:**
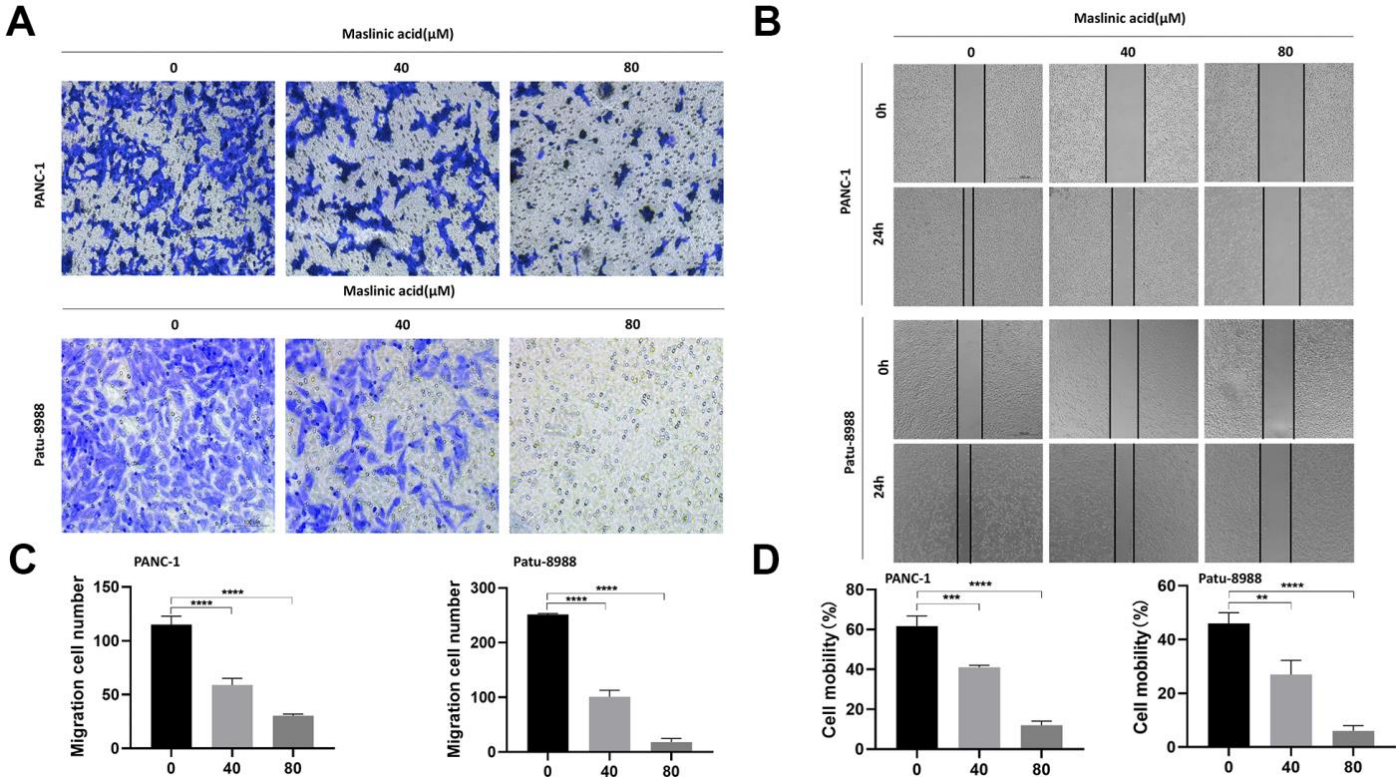
**MA-mediated inhibition of the migration of PANC-1 and Patu-8988 cells.** (**A**) Transwell chamber assay analysis of the effect of MA (0, 40, 80 μM) on the migration of PANC-1 and Patu-8988 cells. (**B**) Wound healing assay analysis of PANC-1 and Patu-8988 cells incubated with MA (0, 40, 80 μM) for 24 h. (**C**) The quantification results of migration cell number. (**D**) The quantification results of cell mobility. Data are representative of three independent experiments and expressed as mean ± SD. **p < 0.01, ***p < 0.001, ****p < 0.0001. Scale bar =100μm.

**Figure 5:**
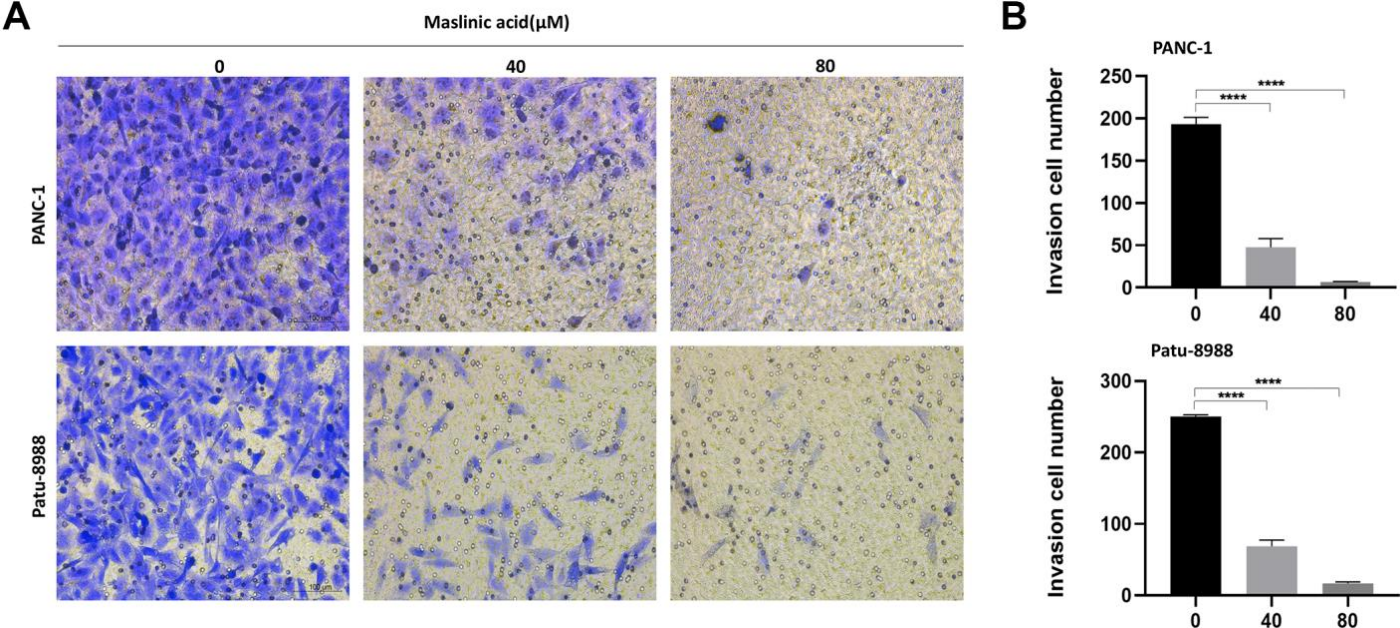
**MA-mediated inhibition of the invasion of pancreatic cancer cells.** (**A**) Transwell assay following PANC-1 and Patu-8988 cells incubated with MA (0, 40, 80 μM) for 24 h demonstrating that MA significantly inhibited invasion of PANC-1 and Patu-8988 cells of pancreatic cancer. in a concentration-dependent manner. (**B**) The quantification results of invasion cell number. Data are representative of three independent experiments and expressed as mean ± SD. ****p < 0.0001. Scale bar =100μm.

We investigated the effect of MA on cell migration and invasion using transwell migration assay and wound healing assay, respectively. The transwell cell migration assay results demonstrated that MA significantly reduced the number of PCCs migrating through the transparent PET membrane (Figure 4A, 4C). At the same time, treatment with MA saw a significant decrease in the velocity of PCCs migrating into the wound gap area (Figure 4B, 4D). The invasive capacity of PANC-1 and Patu-8988 cells was suppressed significantly after exposure to MA (Figure 5A, 5B).

### RNA-seq analysis of PANC-1 cells with or without MA treatment

The functional mechanism of MA on PCCs was investigated by RNA-seq of PCCs treated with or without MA (40 μM) for 24 h. The data of quality control of the transcriptome samples is illustrated in Supplementary Figure 1. Using the fold Change >= 2 or <= 0.5 and P-value < 0.05 as the screening criteria, 238 differentially DEGs in RNA-seq were identified between MA treated PANC-1 and PANC-1 cells, comprising 79 up-regulated genes and 159 down-regulated genes (Supplementary Table 1).

### GO and KEGG pathway enrichment analyses of DEGs

**Figure 6:**
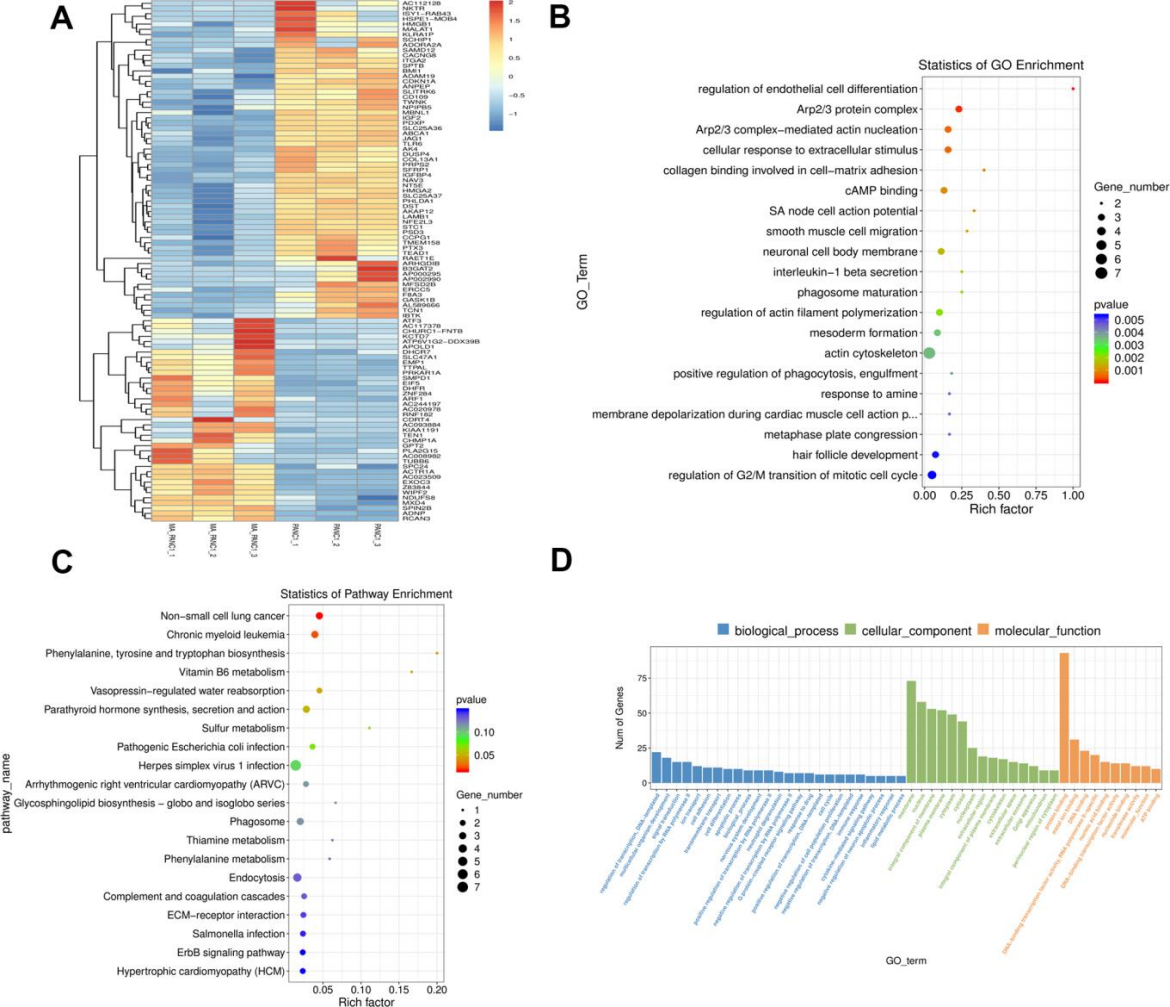
**GO and KEGG pathway enrichment analysis of DEGs between PANC-1 and MA treated PANC-1.** (**A**) The cluster heat map of the first 100 DEGs in the PANC-1 and MA PANC-1 datasets. The abscissa indicates the number of samples, whereas the ordinate indicates DEGs. The histogram in the upper right corner represents the color level; each rectangle corresponds to the expression value of a sample. (**B**, **C**) GO enrichment analysis and KEGG pathway enrichment analysis of DEGs in PANC-1 cells post MA treatment. The results showed the GO Term and pathway of the top 20 enriched significantly in the form of a scatter plot. (**D**) GO term of Top 25, Top15, Top 10. According to the number of differential genes annotated to GO Term, they are arranged in descending order, showing the distribution of the number of significantly different genes in GO Term enriched in biological processes, cell components, and molecular functions.

The expression of DEGs in MA treated PANC-1 and PANC-1 cells was explored by clustering the genes according to the similarity of gene expression profiles of the samples. The top 100 genes with the smallest p-value were displayed in a heat map, in which, the abscissa is the sample and the ordinate represent the differentially expressed genes screened out. Different gene expression levels were indicated by different colors. For instance, the colors from blue to white to red denote expression levels from low to high, where blue indicates lowly expressed genes and red indicates highly expressed genes (Figure 6A).

The function of DEGs was further elucidated via GO and KEGG pathway enrichment analyses. The GO enrichment analysis revealed a strong association of the DEGs in the MA group with the regulation of endothelial cell differentiation, Arp2/3 protein complex, Arp2/3 complex-mediated actin nucleation, cellular response to extracellular stimulus, and collagen binding involved in cell-matrix adhesion molecular function (Figure 6B). The KEGG pathway enrichment analysis revealed a close association of the DEGs in the MA group with non-small cell lung cancer, chronic myeloid leukemia, and phenylalanine, tyrosine, and tryptophan biosynthesis. (Figure 6C). The DEGs were categorized based on cellular components (CC), biological processes (BP), and molecular functions (MF). In the biological process group, DEGs were mainly enriched in the regulation of transcription, DNA-templated, multicellular organism development, and signal transduction. GO cell component analysis revealed significant enrichment of DEGs in the membrane, nucleus, and integral component of the membrane. For molecular function, the DEGs were enriched in protein binding, mental ion binging, and DNA binding (Figure 6D).

### Proteomics analysis of differentially expressed proteins

In addition to transcriptome analysis, gene expression at the translational level was evaluated via TMT and LC-MS/MS analyses. The data of quality control of the proteomic samples is shown in Supplementary Figure 2. Following quality validation, 38,473 (27,808 matched) spectra were obtained, of which 38,966 peptides (36,544 unique peptides) and 5,450 were identified proteins (4,636 quantifiable proteins) were detected.

Complete mass spectrometry data of these protein are provided in Supplementary Table 2. In total, 55 DEPs (39 DEPs up-regulated and 16 DEPs down-regulated) were identified (fold change >= 1.2 or <=0.8333 and p value < 0.05) (Supplementary Table 3).

### GO and KEGG pathway enrichment analyses of DEPs

**Figure 7:**
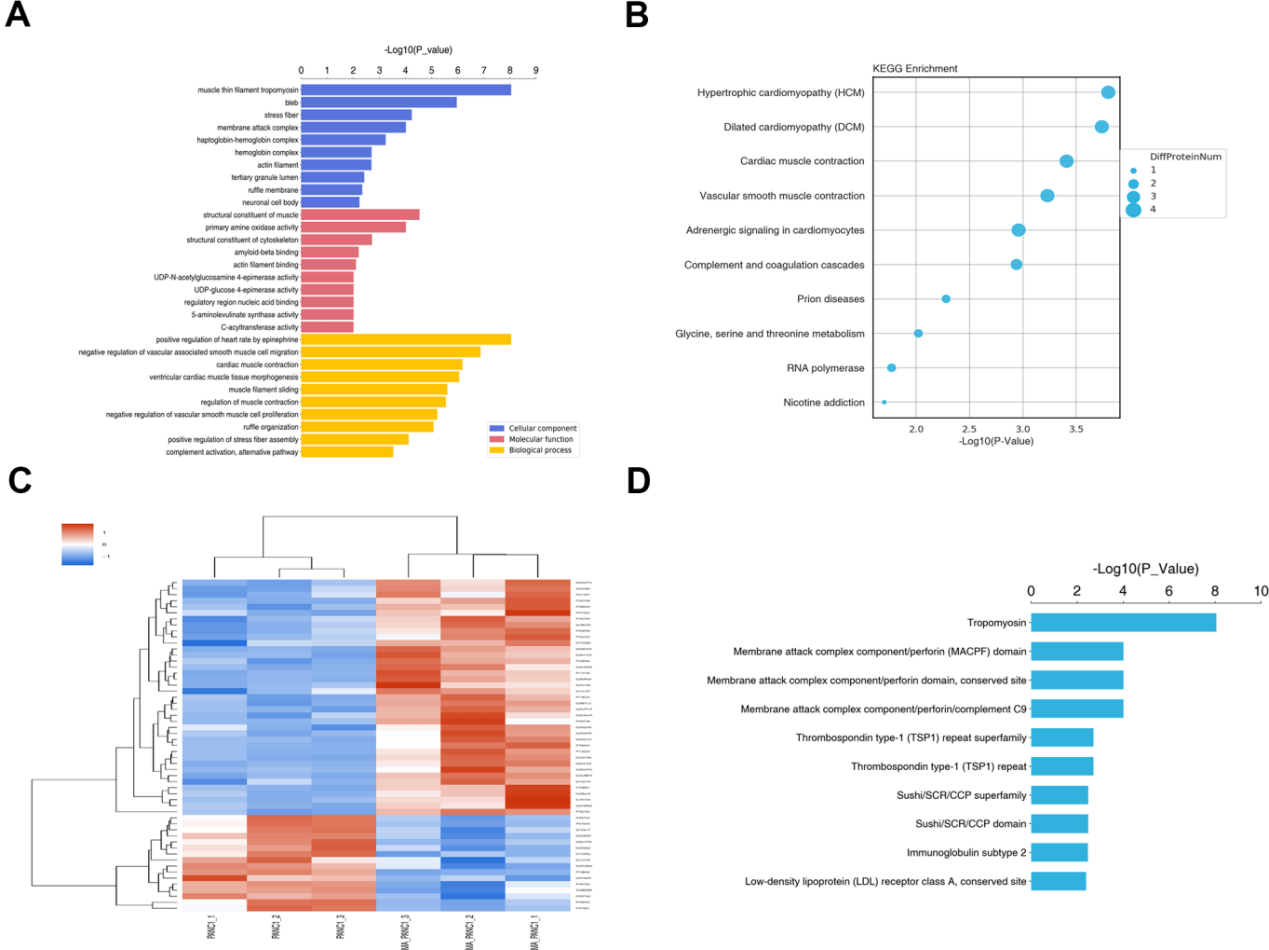
**GO and KEGG pathway enrichment analysis of DEPs between PANC-1 and MA treated PANC-1.** (**A**) Go enrichment analysis of DEPs in PANC-1 cells post MA treatment. The results showed the GO Term of the top 10 enrichment significance. (**B**) The top 10 enriched pathways for DEPs. (**C**) Differential protein group Cluster analysis. (**D**) The top 10 significantly enriched protein domain classification statistics histogram.

The functions of these DEPs were explored through GO enrichment analysis. The DEPs were categorized based on cellular components (CC), biological processes (BP), and molecular functions (MF). In the biological process group, DEPs were mainly enriched in positive epinephrine-mediated regulation of heart rate, negative regulation of vascular-associated smooth muscle cell migration, and cardiac muscle contraction. GO cell component analysis revealed significant enrichment of DEPs in the muscle-thin filament tropomyosin, bleb, and stress fiber. For molecular function, the DEPs were enriched in a structural constituent of muscle, primary amine oxidase activity, and structural constituent of the cytoskeleton (Figure 7A).

For functional classification, the DEPs were mapped to the KEGG pathways using KOALA. Results showed that proteins in the MA group were largely associated with hypertrophic cardiomyopathy (HCM), dilated cardiomyopathy (DCM), and cardiac muscle contraction (Figure 7B). The hierarchical clustering heat map of the DEPs is shown in Figure 7C. Structural domains are the units of protein structure, function, and evolution. As such, research on protein domains is of great significance to understand the biological functions and evolution of proteins. Using the Interpro database, we analyzed the annotation and enrichment of the functional domains of the differential proteins (Figure 7D). The top 3 categories with the most significant enrichment were tropomyosin, membrane attack complex component/perforin domain, membrane attack complex component/perforin domain, and conserved site (Figure 7D).

### Verification of the expression levels of DEGs and DEPs

**Figure 8:**
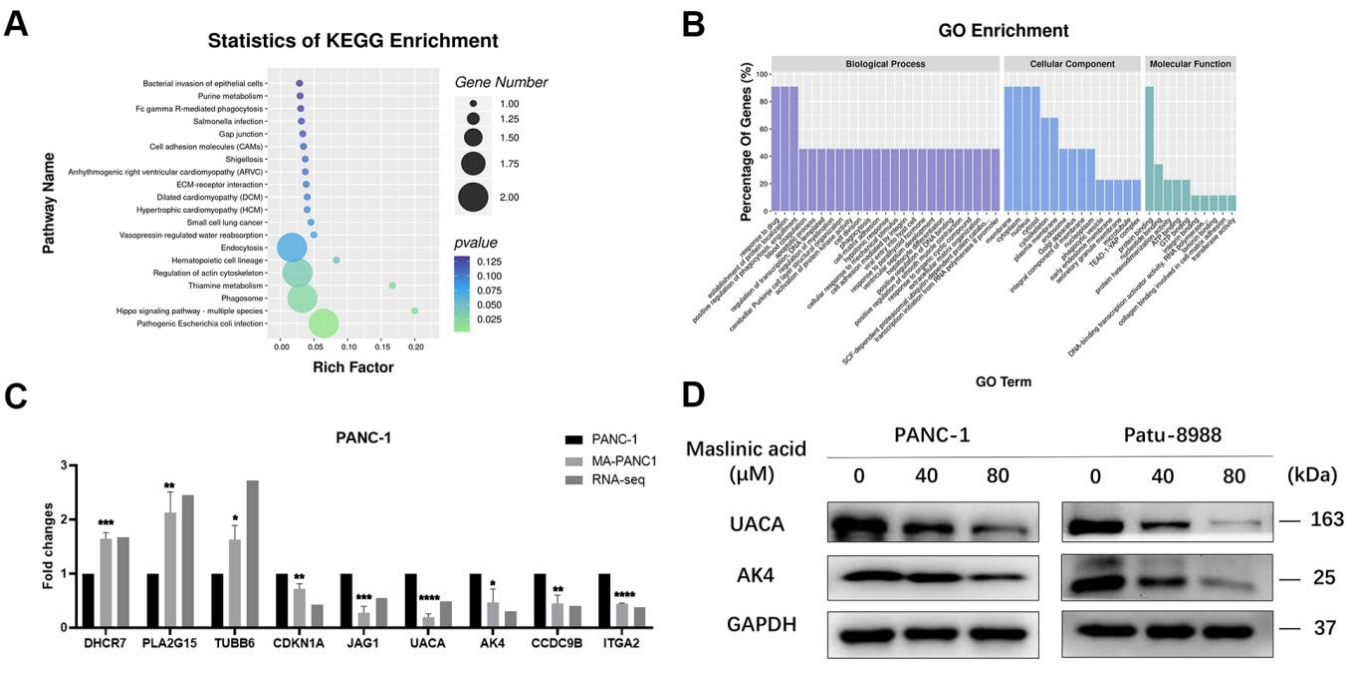
**Association analysis of differential genes and differential proteins.** (**A**) The top 20 enriched pathways for DEGs. (**B**) Top 25, top15, and top10 GO Terms according to the number of different genes annotated to GO terms in descending order. (**C**) QRT-PCR analysis of DEGs in PANC-1 and MA-treated PANC-1 cells. (**D**) Western blot analysis of the expression of UACA and AK4 in MA-treated PANC-1 and Patu-8988 cells. Data are presented as fold change and as mean ± SD of three independent experiments. ***p < 0.001, ****p <0.0001.

To validate the transcriptome data, 9 genes (DHCR7, PLA2G15, TUBB6, CDKN1A, JAG1, UACA, AK4, CCDC9B, ITGA2) were selected randomly among the DEGs, and their expression levels were verified by qRT-PCR. QRT-PCR results showed a similar expression trend to the transcriptome analysis, providing evidence on the reliability of the transcriptome sequencing results (Figure 8C). For proteomics verification, 2 genes (UACA and AK4) identified to be closely related to cancer, according to the results of association analysis, were subjected to western blot. Similar expression trends similar to those of proteomics results were revealed by western blot (Figure 8D).

Housekeeping genes for basic cell maintenance are also suggested to maintain constant expression levels in all cells, under all conditions [14]. Our analysis revealed that housekeeping proteins, including ACTB, PGK1, RPS18, RPS27A, RPL11, RPL19, RPL32, NONO, ARHGDIA, TUBA1B, TUBA1C, TUBB4B, and HSP90B1 were stably expressed in both the control and MA groups (Supplementary Figure 2C), demonstrating the reliability of the proteomic sequencing results [15].

### Correlation analysis of the transcriptome and proteome data in the MA treatment group

As the translation product of mRNA, proteins exert specific functions. Correlation analysis between transcriptome data and proteome data based on the translation relationship between mRNA and protein yielded 24 genes overlapping between the transcriptome and the proteome (Table 1). The related genes between mRNA level and protein level were categorized according to cellular components (CC), biological processes (BP), and molecular functions (MF). In the biological process group, DEGs were mainly enriched in the regulation of the G2/M transition of the mitotic cell cycle, response to drugs, and neutrophil degranulation. GO cell component analysis revealed significant enrichment of DEGs in cytosol, cytoplasm, and membrane. Furthermore, the KEGG pathway enrichment analysis revealed that DEGs in the MA group were mainly related to pathogenic *Escherichia coli* infection, Hippo signaling pathway, phagosome, and thiamine metabolism (Figure 8A). For molecular function, the DEGs were enriched in protein binding, nucleotide-binding, and ATP binding (Figure 8B). The 24 genes can be categorized into four groups according to the correlation between mRNA and protein levels. The first category comprises 6 genes (MPZL1, CBLL1, ATXN7, ADNP, STBD1, TOMM6) whose mRNA level and protein level are up-regulated. The second category comprises, 3 genes (TUBB6, ACTR1A, PRKAR1A) whose mRNA level is up-regulated but the protein level is down-regulated. The third category comprises 10 genes (UACA, ITGA2, TEAD1, RAB31, AK4, CCDC9B, DCTN5, KIF14, GULP1, LEPROT) whose mRNA level and protein level are down-regulated. The fourth category comprises 5 genes (NKTR, TWNK, CCPG1, CENPF, ARPC5) whose mRNA level is down-regulated but the protein level is up-regulated. The results showed that the expression levels of the second and fourth category of genes was opposite. This may be ascribed to several levels of regulation, such as RNA and protein turnover, post-translational modifications, protein conformational changes, and proteolysis.

**Table 1:** Representative genes differentially expressed in the PANC-1 and MA treated PANC-1 at the mRNA and protein levels.

**mRNA information**	**Protein information**	**Function description**	**mRNA sig p-value**	**Protein sig p-value**	**Regulation type (mRNA/protein)**
TUBB6	Q9BUF5	GTPase activity	YES	YES	Up/ Down
ACTR1A	P61163	Microtubule organizing center	YES	YES	Up/ Down
UACA	Q9BZF9	Apoptotic signaling pathway	YES	YES	Down/ Down
NKTR	P30414	Nucleoplasm	YES	NO	Down/ Up
TWNK	Q96RR1	Protein hexamerization	YES	NO	Down/ Up
ITGA2	P17301	Nucleus	YES	YES	Down/ Down
CCPG1	Q9ULG6	Membrane	YES	NO	Down/ Up
TEAD1	P28347	Nucleus	YES	NO	Down/ Down
CENPF	P49454	Nuclear matrix	YES	NO	Down/ Up
MPZL1	O95297	Structural molecule activity	YES	NO	Up/ Up
PRKAR1A	P10644	Activation of protein kinase A activity	YES	NO	Up/ Down
CBLL1	Q75N03	Negative regulation of cell adhesion	YES	YES	Up/ Up
RAB31	Q13636	GTPase activity	YES	NO	Down/ Down
AK4	P27144	Nucleoside diphosphate kinase activity	YES	YES	Down/ Down
ATXN7	O15265	Nuclear matrix	YES	NO	Up/ Up
CCDC9	Q6ZUT6	RNA binding	YES	YES	Down/ Down
DCTN5	Q9BTE1	Cytoplasm	YES	NO	Down/ Down
KIF14	Q15058	Establishment of protein localization	YES	NO	Down/ Down
ARPC5	O15511	Smooth muscle cell migration	YES	NO	Down/ Up
ADNP	Q9H2P0	Short-term memory	YES	NO	Up/ Up
STBD1	O95210	Autophagy	YES	NO	Up/ Up
GULP1	Q9UBP9	Protein binding	YES	NO	Down/ Down
LEPROT	O15243	Integral component of membrane	YES	YES	Down/ Down
TOMM6	Q96B49	Mitochondrial outer membrane translocase complex	YES	NO	Up/ Up

### Overexpression of UACA and AK4 abolishes MA- mediated anti-tumor effect

**Figure 9:**
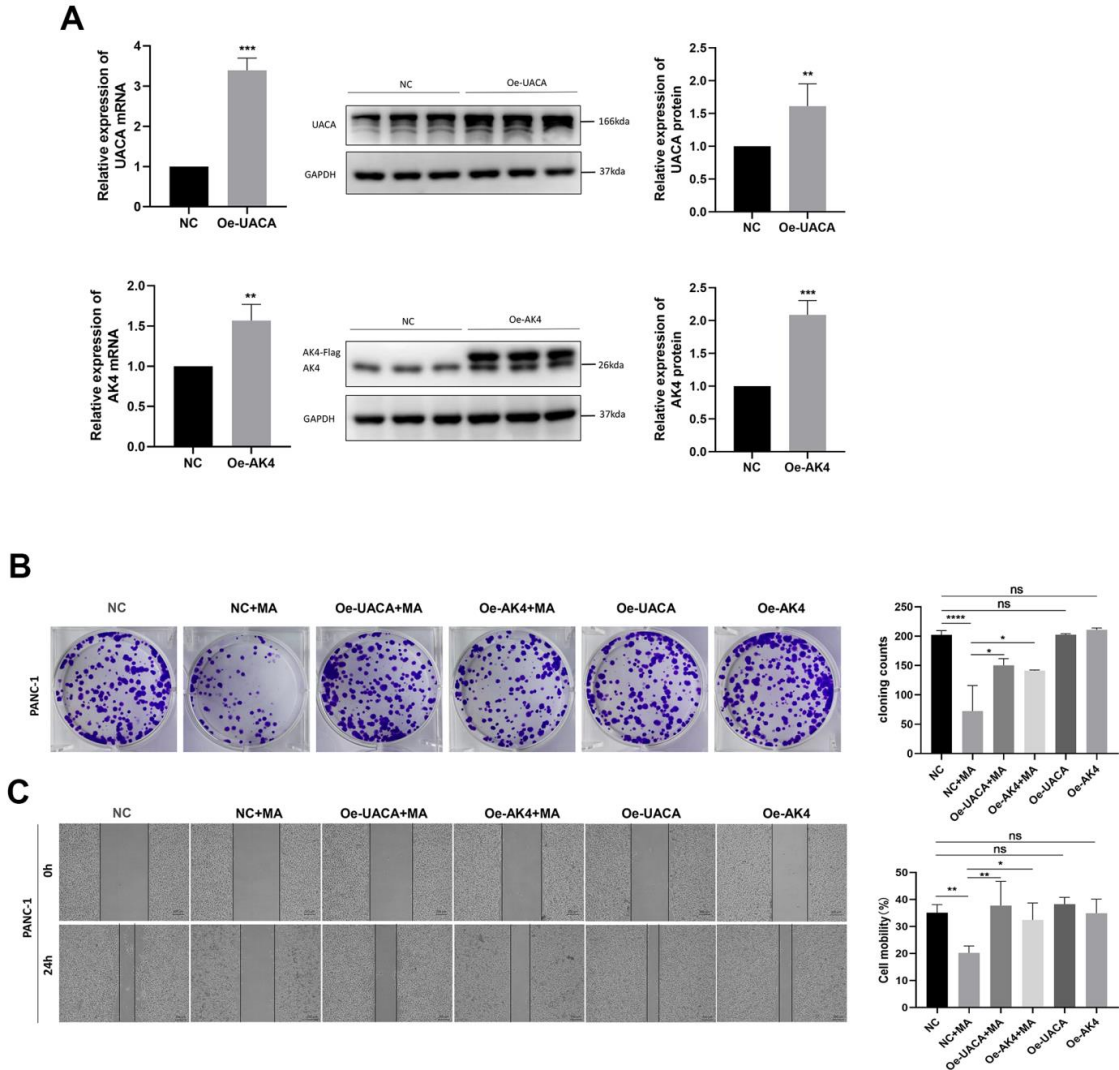
**Overexpression of UACA and AK4 abolishes the MA-mediated anti-tumor effect.** (**A**) QRT-PCR and western blot analysis of the expression of UACA and AK4 in NC, oe-UACA, oe-AK4 groups of PANC-1 cells. (**B**) Colony formation assay analysis of cell proliferation in NC, NC+MA (40μM MA treated for 24 h), oe-UACA+MA (40μM MA treated for 24 h), oe-AK4+MA (40μM MA treated for 24 h), oe-UACA, oe-AK4 groups of PANC-1 cells. (**C**) Wound healing assay analysis of cell migration in NC, oe-UACA+MA (40μM MA treated for 24 h), oe-AK4+MA (40μM MA treated for 24 h), oe-UACA, oe-AK4 groups of PANC-1 cells. Bar =200 μm. Data are presented as fold change and as mean ± SD of three independent experiments. *p <0.05, **p < 0.01, ****p < 0.0001.

The transfection efficiency of UACA and AK4 overexpression of PANC-1 cells was verified through qRT-PCR and western blot analysis (Figure 9A). Results demonstrated markedly higher levels of UACA or AK4 in cells transfected with pcDNA3.1(+)-UACA-Flag or pcDNA3.1(+)-AK4-Flag compared to cells transfected with control pcDNA3.1(+). The colony-forming ability of the oe-UACA and oe-AK4 groups did not change as compared to the NC group (Figure 9B). These results demonstrate that overexpression of the UACA or AK4 gene would not affect the proliferation of pancreatic cancer cells. Besides, results of the colony formation test demonstrated that overexpression of UACA or AK4 could abolish the anti-proliferative effect of 40μM MA treated for 24 h (Figure 9B). Wound healing experiments further confirmed that overexpression of UACA or AK4 does not influence the migration ability of pancreatic cancer cells (Figure 9C). At the same time, overexpression of UACA or AK4 was found to abolish the anti-migration effect realized by treatment with MA (40μM) for 24 h. These data validate UACA and AK4 genes as key molecular targets for the anti-pancreatic cancer effect of MA.

## DISCUSSION

Recent evidence indicates that large quantities of bioactive triterpenoids are cytotoxic to multiple tumor cells, including breast cancer, lung cancer, and colorectal cancer [16–18]. In this study, we used PANC-1 and Patu-8988 as cell models to study the effects of MA (a triterpenoid compound isolated from olive peel pomace) on the proliferation, apoptosis, migration, and invasion of PCCs. The chemical structure of MA is shown in Figure 1A. Our analysis confirmed the anti-proliferative effect of MA on PANC-1 and Patu-8988 cells. MA inhibited the colony formation of pancreatic cancer cells and the expression of proliferation-related protein Ki67 and induced PCC apoptosis, significantly. Exploring the DEGs and DEPs in PANC-1 cells after the treatment of MA allowed for the investigation of the specific mechanism of MA. The key genes and proteins related to the anti-cancer activity of MA were further elucidated through transcriptomics and proteomics analysis. The findings provide strong evidence for the role of MA as a potential complementary treatment for pancreatic cancer.

High-throughput analysis of the gene expression of PANC-1 cells treated with MA enabled for screening of key genes and proteins related to the role of MA in inhibiting pancreatic cancer. Of the 238 DEGs and 55 DEPs identified in MA treated PANC-1 cells, 79 genes and 39 proteins were up-regulated, whereas the remaining 159 genes and 16 proteins were down-regulated. KEGG enrichment analysis demonstrated that the differential genes were mainly involved in the pathways of non-small cell lung cancer, chronic myeloid leukemia, phenylalanine, tyrosine, and tryptophan biosynthesis. There is previous evidence that non-small cell lung cancer and chronic myeloid leukemia are crucial KEGG pathways in pancreatic cancer [19]. Consistent findings from our sequencing results indicated that MA plays a part in the regulation of pancreatic cancer cells and is closely related to the above pathways. Moreover, the enrichment of phenylalanine, tyrosine, and tryptophan biosynthesis provide more evidence that MA treatment tunes the amino acid metabolism process of pancreatic cancer cells. Through GO enrichment analysis, the differential genes were revealed to be concentrated in signaling pathways related to the regulation of endothelial cell differentiation, collagen-binding involved in cell-matrix adhesion, regulation of actin filament polymerization, actin cytoskeleton, and regulation of G2/M transition of the mitotic cell cycle. These data supported the view that MA has potent inhibitory effects on the proliferation, migration, invasion, and other malignant processes of pancreatic cancer cells by regulating the cytoskeleton and G2/M cell cycle.

Although a consensus in the literature demonstrates a direct correspondence between the transcription of the mRNA and the protein and the translated protein, the stability and abundance of the mRNA are 5 times and 900 times lower than that of the protein, with a higher dynamic range [20]. Therefore, we, herein, focused on the overlapping genes between the transcriptome and the proteome. Screening through the association analysis of transcriptome and proteome data yielded 24 genes that overlap between transcriptome and proteome, among which 8 genes (ie NKTR, TWNK, CCPG1, CENPF, ARPC5, PRKAR1A, TUBB6, and ACTR1A) showed opposite trends at the transcription level and protein level. This phenomenon may ay be is possibly ascribe to various factors, including different half-lives, post-transcriptional and translational changes [21]. Of the remaining 16 genes, 6 genes (up-regulated, n=1; down-regulated, n=5) exhibited significant differences in the transcription and translation levels of the treatment group. On the basis of previous research, two genes, UACA and AK4 that may be associated with the anti-tumor mechanism of MA, were screened and discussed.

Correlation analysis results demonstrated that MA treatment downregulated the expression of uveal autoantigen with coiled-coil domains and ankyrin repeats (UACA) gene in both the transcriptome and proteome. qRT-PCR and western blot results were consistent with the sequencing results. UACA was originally identified as a novel autoantigen in patients with pancreatitis, and further evidence demonstrated a higher incidence of IgG anti-UACA antibodies in Vogt-Koyanagi-Harada (VKH) patients than that in healthy controls [22]. Compelling evidence indicates that UACA is an oncogene and is up-regulated in HCC. On the other hand, UACA knockdown, potentially regulated by hypoxia, was found to suppress the malignant behavior of HCC [23]. The immunohistochemical results of Ravshan et al. showed that UACA expression in human prostate cancer specimens increased by 3 times or more. Similarly, significantly higher expression of UACA was reported in lung adenocarcinoma and squamous cell carcinoma as compared to normal lung specimens [24]. In this view, inhibition of UACA expression can enhance the sensitivity of cancer cells to apoptosis. In the present study, MA inhibited the expression of UACA and is suggested to suppress pancreatic cancer cells by down-regulating the UACA gene. As far as we know, the role of UACA in pancreatic cancer has not been reported. Evidence from our laboratory indicates that UACA overexpression does not affect the proliferation and migration ability of pancreatic cancer cells. Intriguingly, overexpression of UACA abolished the pancreatic antitumor activity of MA, providing evidence that MA exerts anti-pancreatic tumor activity by targeting UACA.

Adenylate kinase (a nucleoside monophosphate kinase) mediates various cellular functions, as among them, the regulation of energy metabolism homeostasis [25, 26]. Adenylate kinase 4 (AK4) is a member of adenylate kinases, expressed in the mitochondrial matrix [27]. Mounting evidence shows that, although AK4 has no enzymatic activity *in vitro*, it can retain the potential to bind to nucleotides; as such, it interacts with the ADP/ATP transporter to promote cell survival and proliferation [28, 29]. Studies have shown that AK4 plays a role in the occurrence and development of cancer, which is why it is a potential target for anti-cancer therapy. For instance, the expression level of AK4 influences the sensitivity of anticancer drugs by regulating the activity of mitochondria [30]. By comparing gene expression profiles, studies have reported significantly overexpressed AK4 levels in highly aggressive tumors [30, 31]. However, these works are yet to report the role of AK4 in pancreatic cancer. The present study revealed that overexpression of AK4 does not affect the proliferation and migration ability of pancreatic cancer cells but could abolish the pancreatic antitumor activity of MA. Therefore, MA exerts anti-pancreatic tumor activity by targeting AK4.

This study has some limitations. First, multiple possible MA targets have been revealed through association analysis; however, only the genes that are closely related to cancer were explored. Therefore, other genes need to be verified in further studies. Also, the mechanism of action between UACA, AK4 molecules, and downstream pathways warrants further exploration.

In summary, MA has promising inhibitory effects on the proliferation, migration, invasion of pancreatic cancer cells, and can induce apoptosis in these cells. Mechanistically, MA exerts an anti-pancreatic cancer effect by suppressing the expression of key genes, including UACA and AK4. More evidence demonstrates that overexpression of UACA and AK4 eliminates the inhibitory effect of MA-mediated PCC proliferation and migration. Therefore, MA holds promise as a potential chemopreventive drug for pancreatic cancer management.

## MATERIALS AND METHODS

### Cell culture and treatment

Human pancreatic cancer cell lines PANC-1 and Patu-8988 were purchased from the American Type Culture Collection (ATCC, Manassas, VA, USA). PANC-1 and Patu-8988 cells were maintained in Dulbecco’s Modified Eagle Medium (Gibco, Grand Island, NY, USA), supplemented with 10% fetal bovine serum (Gibco, Grand Island, NY, USA), streptomycin (100 μg/ml), and penicillin (Invitrogen) (100 IU/ml) at 37° C in a 5% CO_2_ atmosphere. PCCs were treated with 40 or 80 μM MA (CAS No.: 4373-41-5, Formula: C_30_H_48_O_4_, purity≥98.0%, Haoyuan Biotechnology, Shanghai, China) or 0.1% DMSO.

### Cell transfection

Using jetPRIME Transfection Reagent (Polyplus, New York, NY, USA), cell transfection was performed following the manufacturer’s protocol. pcDNA3.1(+)-UACA-Flag (Oe-UACA), pcDNA3.1(+)-AK4-Flag (Oe-AK4), and pcDNA3.1 negative control (Oe-NC) vectors were acquired from PPL (Public Protein/Plasmid Library, Nanjing, China). PANC-1 cells were transfected with Oe-UACA, Oe-AK4, and Oe-NC, respectively, for 24h. The transfection efficacy was then detected by qRT-PCR and western blotting.

### Real-time cellular analysis

The label-free real-time cell analysis (RTCA) system (Roche, Penzberg, Germany) was employed to assess cell proliferation. PANC-1 and Patu-8988 cells were seeded on a cell culture E16-Plate (ACEA Biosciences, San Diego, CA, USA) at a density of 5×10^3^ cells/well. After the cells adhered, MA at different treatments (0, 40, and 80 μM) were added to the wells on separate plates. The cell growth index was monitored every 15 min for 3 days.

### Colony formation assay

PANC-1 and Patu-8988 cells (1000 cells per well) were incubated in 6-well plates, allowed to attach, and treated with MA or 0.1% DMSO for 24 h. The old medium was replaced by DMEM without MA. Cells were incubated for 2 weeks, and the resultant colonies were fixed and stained with crystal violet. Images of the colonies were captured using digital camera and the number of colonies was counted. All experiments were conducted in triplicates.

### Wound healing assay

PANC-1 and Patu-8988 cells were incubated in a 6-well plate at 37° C to create a confluent monolayer. Cell monolayers were scraped using a sterile micropipette tip and washed with 0.01M PBS once, and a medium containing 0, 40, and 80 μM MA was added. Images were captured with an inverted microscope every 12 h. All experiments were conducted in triplicates.

### Transwell invasion and migration assay

For cell migration assay, 200 μL of serum-free media including 1×10^5^cells with MA (0,40,80 μmol/L) was added to the upper chamber of a transwell (8.0 μm pore size, Corning Incorporated, Kennebunk, ME, USA), coated with Matrigel reduced by growth factor (356230, Corning, NY, USA), and 600 μL of media with 10% FBS was added to the lower chamber. After 24 h, the chambers were fixed with paraformaldehyde and stained with crystal violet for 30 min. Cells that did not pass through the Matrigel were wiped away. Images of 3-5 fields of chambers selected randomly were captured using a fluorescence microscope, and cells were counted.

The procedure of the transwell migration assay was performed in the same way as that of the invasion assay, except that the upper compartment was not coated with Matrigel.

### Immunofluorescence

PANC-1 and Patu-8988 cells were cultured in six-well plates containing glass coverslips, fixed in 4% paraformaldehyde (Sigma-Aldrich, St. Louis, MO, USA) for 15 min at 4° C. Next, 0.1% Triton X-100 (Sigma-Aldrich, St. Louis, MO, USA) was permeated for 10 min, and cells were washed with PBS and blocked in 5% bovine serum albumin (BSA) for 1 h at room temperature. Cells were incubated with primary antibody Ki67 (1: 200, Cell Signaling Technology, 12075) at 4° C overnight. Cells were then washed three times in PBS, and incubated with Cora®Lite 488 secondary antibodies (1:400, Proteintech, Cat No.:SA00013-2) for 1 h at 37° C. Cells were washed three times, and the nuclei were stained with 4′, 6-diamino-2-phenylindole (DAPI, Sigma-Aldrich, St. Louis, MO, USA). Ki67-positive cells were examined via immunofluorescence microscopy.

### Flow-cytometry detection of apoptosis

PANC-1 and Patu-8988 cells were treated with MA at 0, 40, and 80 μM for 24 h. Annexin V-FITC Apoptosis Detection Kit (BD) was used for apoptosis analysis according to the manufacturer’s protocol. Briefly, cells were collected by centrifugation and resuspended in a binding buffer. Annexin V-FITC and propidium iodide (5 μl each), were added followed by incubation at room temperature in the dark for 15 min. Cell apoptosis was analyzed by a FACSCalibur flow cytometer (BD Biosciences, Franklin Lakes, NJ, USA). Data were analyzed in the FlowJo software (version 10.0.7).

### Quantitative reverse transcriptase-PCR analysis

Total RNA was isolated from PCCs using TRIzol reagent (Invitrogen, Carlsbad, CA, USA). CDNA was synthesized from total RNA using the reverse transcription kit (Thermo Fisher Scientific) following the manufacturer’s instructions. Thermocycling conditions were: 40 cycles each with 95° C for 10 s for denaturation, 60° C for 20 s for annealing, and 72° C for 30 s for extension, performed in ABI PRISM 7500FAST PCR Sequence Detection System (Thermo Fisher Scientific). The fold changes of target genes between the experiment group and the control group were calculated according to the 2^−ΔΔCt^ method. All quantitative RT-PCR reactions were run in three independent experiments. The following primer pairs were used:

DHCR7 F: 5′- GCTGCAAAATCGCAACCCAA-3′, R: 5′-GCTCGCCAGTGAAAACCAGT-3′.

PLA2G15 F: 5′- CCGAAAGCTACTTCACAATCTGG-3′, R: 5′-CAGGGACACGTACATCCACAC-3′.

TUBB6 F: 5′- TGGTGGACTTAGAGCCAGG-3′, R: 5′-CCCTTTCGCCCAGTTGTTC-3′.

CDKN1A F: 5′- TGTCCGTCAGAACCCATGC-3′, R: 5′-AAAGTCGAAGTTCCATCGCTC-3′.

JAG1 F: 5′- GTCCATGCAGAACGTGAACG-3′, R: 5′-GCGGGACTGATACTCCTTGA-3′.

UACA F: 5′-TCAAGGAGCAAGCACATAAC-3′, R: 5′-GCTGTCATTTTCCAGTAGCA-3′.

AK4 F: 5′-AGGGGAGATTCACTTCCTG-3′, R: 5′-CCAAAGAGATGGGCACAC-3′.

CCDC9B F: 5′- CCCACCATTGCTCCCTGAT-3′, R: 5′-GCTTCCCTCCTGACCTTCC-3′.

ITGA2 F: 5′-CAGCAACCAAAACAAAAGG-3′, R: 5′-CAGGGAGAATGATGCAAAA-3′.

GAPDH F: 5′- CCTTCCGTGTCCCCACT-3′, R: 5′-GCCTGCTTCACCACCTTC-3′.

### Western blot analysis

PANC-1 and Patu-8988 human pancreatic cancer cells were seeded in a 6-well plate and treated with different concentrations of MA for a scheduled time. Total proteins were isolated from PANC-1 and Patu-8988 cells. Protein concentrations were determined by a BCA protein assay kit (Beyotime Biotechnology, Shanghai, China). Total proteins (30 μg) from each sample were separated in SDS-PAGE and transferred to an activated PVDF membrane (IPVH00010, Millipore, Massachusetts, USA). After that, proteins were blocked with 5% skim milk in TBST for 1 h at room temperature, and then incubated overnight at 4° C with a primary antibody. After three washes (10 min each) in TBST, membranes were incubated with the secondary antibodies for a further 1 h at room temperature. The protein bands were visualized via chemiluminescence detection on autoradiographic film. Proteins were quantified by measuring the intensity of signals using Image-Pro Plus and normalized to that for the GAPDH antibody. The primary antibodies used in this study include: anti-Cleaved Caspase-3 (1:1000, Abcam, ab32042), anti-AK4 (1:1000, Abcam, ab265331), anti-ITGA2 (1:1000, Abcam, ab133557), anti-UACA (1:1000, Proteintech, 25654-1-AP) and anti-GAPDH (1:5000, Cell Signaling Technology, 5174S) antibodies.

### RNA-seq analysis

The data set included 6 PANC-1 cell samples, treated with MA (0μM and 40μM) for 24 h in three biological replicates. Total RNA was extracted from cells and purified using TRIzol reagent (Invitrogen), and shipped to LC-Bio Technology company (Hangzhou, China). RNA-seq library and RNA-seq were constructed by Illumina Novaseq™ 6000 and used for further RNA-SEQ detection and analysis. Differentially expressed mRNAs were selected according to fold change > 2 or fold change < 0.5 and p-value < 0.05 by R package edgeR [32] or DESeq2 [33]. The differentially expressed genes (DEGs) were subjected to Gene Ontology (GO) enrichment and Kyoto Encyclopedia of Genes and Genomes (KEGG) enrichment analysis.

### Tandem mass tag proteomics analysis

### Sample preparation

PANC-1 cells were treated with or without 40 μM MA for 24 h. The cells were collected by centrifugation. Each sample was added to the SDT buffer. The pyrolysate was treated with ultrasound and boiled for 15 min. After centrifugation at 14000g for 40 min, protein concentration was determined using a BCA kit (p0012, Beyotime Biotechnology, Shanghai, China). Protein (20 μg) from each sample was mixed with 6x loading buffer and boiled for 5 min, and separated in 12.5% SDS-PAGE gel. Protein bands were visualized by Coomassie brilliant blue R-250 staining.

### Filter-aided sample preparation (FASP digestion)

Protein (200 μg) of each cell sample was mixed with 30 μl SDT buffer (4% SDS, 100 mM DTT, 150 mM Tris HCl, pH 8.0). Protein ultrafiltration (sartorius, 30 KD) was repeated with UA buffer (8 M urea, 150 mM Tris HCl, pH 8.5). Subsequently, 100 μl iodoacetamide (100 mM IAA in UA buffer) was added, followed by a 30 min incubation in the dark. The protein suspension was digested with trypsin (Promega) (4 μg) in 0.1M DS buffer (40 μl) at 37° C overnight. The resulting peptides were collected as a filtrate. Based on the frequency of tryptophan and tyrosine in vertebrate protein, the peptide content was estimated by UV light spectral density (at 280 nm) using the extinction coefficient of 1.1 of 0.1% (g/l) solution.

### TMT labelling

For each sample, 100 μg of the peptide mixture was labelled using a TMT reagent (Thermo Fisher Scientific) according to the manufacturer’s instructions.

### Peptide fractionation with reversed-phase chromatography

The labeled peptides were fractionated and classified using Agilent 1260 Infinity II HPLC system. The resultant peptide mixture, diluted with buffer A (10mM HCOONH4 and 5% acetonitrile, pH 10.0), was loaded onto a 4.6 mm X 100 mm XBridge Peptide BEH C18 Column. The peptides were eluted at a flow rate of 1 ml/min using the following gradient: 0%–7% buffer B (10mM HCOONH4, 85% ACN, pH 10.0) for 5 min, 7–40% buffer B for 30 min, 40%–100% buffer B for 60 min, 100% buffer B for 65 min. The elution was assessed by UV at 214 nm. Fractions were collected every 1 min and dried.

### Easy nLC

Each fraction was injected for nano LC-MS/MS analysis. The peptide mixture was loaded onto the C18 reverse-phase analytical column (Thermo Fisher Scientific, Acclaim Pep Map RSLC 50um X 15cm, nano viper, P/N164943) in buffer A (0.1% Formic acid) and separated at a flow rate of 300 NL/min, with a linear gradient of buffer B (80% acetonitrile and 0.1% Formic acid). The linear gradient was set as follows: 6% for 3 min, 6-28% for 40 min, 28-38% for 5min,38-100% for 5 min, and 100% for 5 min.

### LC-MS/MS analysis

LC-MS/MS data analysis was conducted as described previously [34].

### TMT data analysis

MS/MS raw data were processed by Mascot engine (version 2.6) embedded into Proteome Discoverer 2.2 (Thermo Fisher Scientific) against a Human protein database (Uniprot_HomoSapiens_20386_20180905). Search parameters included trypsin as the enzyme with 2 missed cleavages. A precursor mass tolerance and MS2 fragment mass tolerance were 10 ppm and 0.05 Da, respectively. We set Carbamidomethyl (C) as a fixed modification, while Oxidation (M) and Acetyl (Protein N-term) were set as variable modifications. The false discovery rate for peptide and protein was set at <1% using a reverse database search strategy [35]. We filtered proteins with expression fold change >1.2 and Student’s t-test, *p*<0.05.

### Bioinformatic analysis

First, the sequence comparison tool NCBI BLAST+ (ncbi-blast-2.3.0+) was employed on the Linux server to compare the target protein collection to the appropriate protein sequence database and retain the front 10 aligned sequences that satisfies E-value<=1e-3 were subjected to subsequent analysis. Secondly, Blast2GO Command-Line was applied to extract the GO entries associated with the target protein set and the highest Bit-Score alignment in the Blast retention results (download link: http://www.geneontology.org). In the KEGG database, KOALA (KEGG Orthology And Links Annotation) software was employed to annotate the target protein set for the KEGG pathway. Fisher’s Exact Test was used to perform GO annotation or KEGG pathway annotation enrichment analysis on the target protein set. For cluster analysis, the matplotlib software was used to classify samples and protein expression in two dimensions (distance algorithm: Euclidean, connection method: Average linkage).

### Statistical analysis

Data are presented as mean ± standard deviation (SD). All experiments were performed in triplicate, independently. GraphPad Prism 8.0 (GraphPad Software, La Jolla, CA, USA) was used for all statistical analyses. Statistically significant differences between groups were analyzed by one-way analysis of variance (ANOVA). Differences with p values less than 0.05 denoted statistical significance.

### Data and materials availability

The materials and data in this study are available upon reasonable request. Raw RNA-seq data were deposited in NCBI’s Gene Expression Omnibus and are accessible through GEO Series accession number GSE183519 (https://www.ncbi.nlm.nih.gov/geo/query/acc.cgi?acc=GSE183519). The mass spectrometry proteomics data have been deposited to the ProteomeXchange Consortium via the PRIDE partner repository with the dataset identifier PXD028303.

## Supplementary Material

Supplementary Figures

Supplementary Table 1

Supplementary Table 2

Supplementary Table 3
