# Supplementary material for: Transcriptome and proteome analysis of the antitumor activity of maslinic acid against pancreatic cancer cells
Source: Aging (Albany NY). 2021 Oct 12;13(19):23308–27. doi: 10.18632/aging.203623 (PMC8544341; doi:10.18632/aging.203623)
Supplement: Supplementary Figures [file aging-13-203623-s001.pdf]

SUPPLEMENTARY FIGURES

A

Sample	Raw Data		Valid Data		Valid Ratio(reads)	Q20%	Q30%	GC content%
	Read	Base	Read	Base				
MA_PANC1_1	39137302	5.87G	38522308	5.78G	98.43	99.98	98.58	51
MA_PANC1_2	44294386	6.64G	43614786	6.54G	98.47	99.98	98.41	52
MA_PANC1_3	40328490	6.05G	39675886	5.95G	98.38	99.98	98.46	50.50
PANC1_1	48795828	7.32G	48019678	7.20G	98.41	99.98	98.39	50
PANC1_2	45533736	6.83G	44840284	6.73G	98.48	99.98	98.63	50.50
PANC1_3	41173482	6.18G	40521082	6.08G	98.42	99.98	98.45	50

B

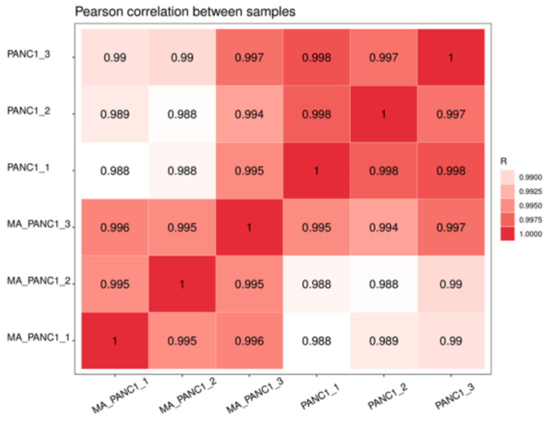

C

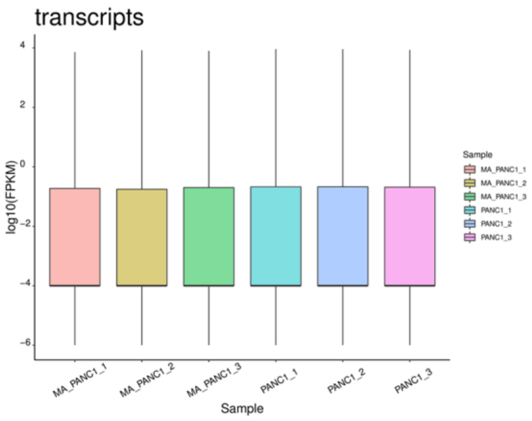

Supplementary Figure 1. (**A**) Correlation analysis results of transcriptome sequencing data. (**B**) Overview of quality control of transcriptome sequencing data. (**C**) Statistical graph of gene expression value distribution of transcriptome sequencing data.

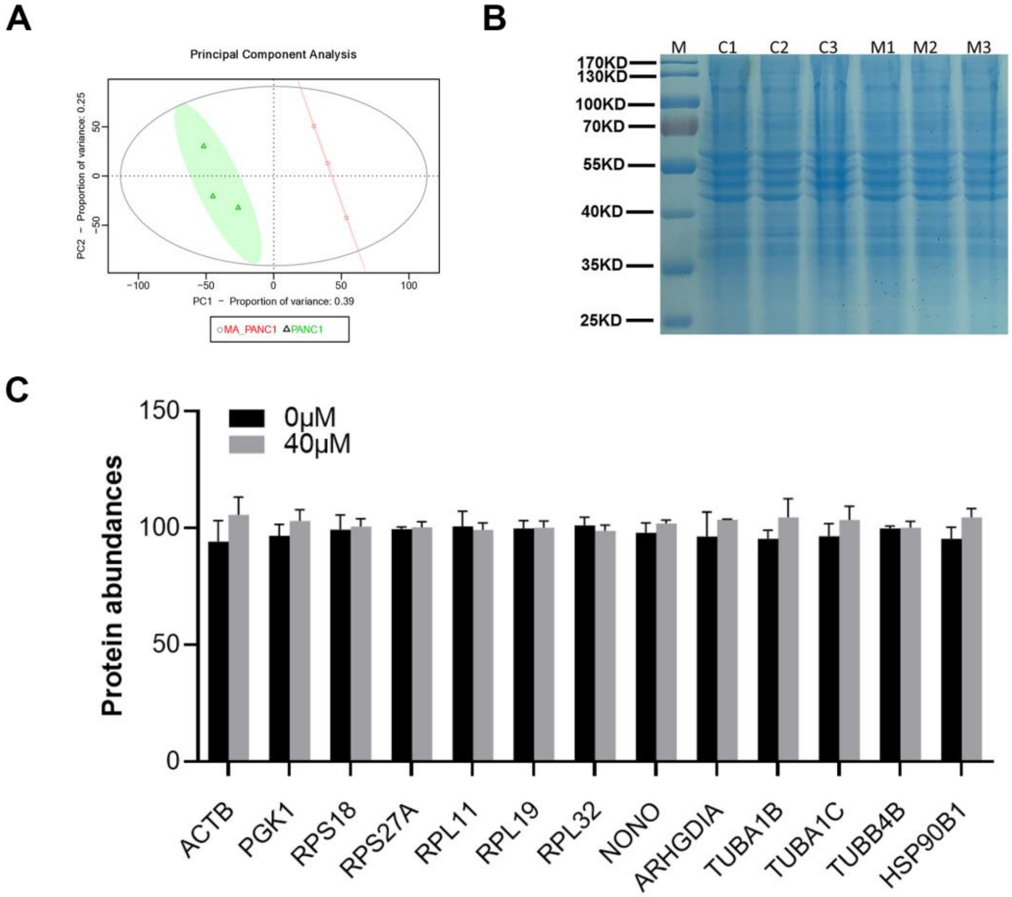

Supplementary Figure 2. (**A**) Principal component analysis of proteomic sequencing data. (**B**) SDS-PAGE test results of proteomic sequencing data. (**C**) Protein abundances of housekeeping genes.
